# Potential of Algae–Bacteria Synergistic Effects on Vegetable Production

**DOI:** 10.3389/fpls.2021.656662

**Published:** 2021-04-12

**Authors:** Yeeun Kang, Minjeong Kim, Changki Shim, Suyea Bae, Seonghoe Jang

**Affiliations:** ^1^World Vegetable Center Korea Office, Wanju-gun, Jeollabuk-do, South Korea; ^2^Organic Agricultural Division, National Institute of Agricultural Sciences, RDA, Wanju-gun, Jeollabuk-do, South Korea

**Keywords:** biofertilizers, combinational application, microalgae, mixed cultures, plant growth-promoting bacteria, vegetables

## Abstract

Modern agriculture has become heavily dependent on chemical fertilizers, which have caused environmental pollution and the loss of soil fertility and sustainability. Microalgae and plant growth-promoting bacteria (PGPB) have been identified as alternatives to chemical fertilizers for improving soil fertility. This is because of their biofertilizing properties, through the production of bioactive compounds (e.g., phytohormones, amino acids, and carotenoids) and their ability to inhibit plant pathogens. Although treatment based on a single species of microalgae or bacteria is commonly used in agriculture, there is growing experimental evidence suggesting that a symbiotic relationship between microalgae and bacteria synergistically affects each other’s physiological and metabolomic processes. Moreover, the co-culture/combination treatment of microalgae and bacteria is considered a promising approach in biotechnology for wastewater treatment and efficient biomass production, based on the advantage of the resulting synergistic effects. However, much remains unexplored regarding the microalgal–bacterial interactions for agricultural applications. In this review, we summarize the effects of microalgae and PGPB as biofertilizing agents on vegetable cultivation. Furthermore, we present the potential of the microalgae–PGPB co-culture/combination system for the environmentally compatible production of vegetables with improved quality.

## Introduction

### Ecological Problems Caused by Chemical Fertilizers in Agriculture

High amounts of chemical fertilizers have been used to obtain high product yields with increased cultivation efficiency in agriculture. However, the excessive use of chemical fertilizers frequently causes severe environmental damage, such as water, soil, and air pollution (Savci, 2012). Moreover, the excessive use of chemical fertilizers leads to soil acidification and hardening, which decrease root vigor with reduced respiration. The population of beneficial microorganisms is also reduced by this practice, resulting in a loss of soil fertility and a high incidence of root diseases (Chandini et al., 2019). In particular, nitrogen (N) fertilizers are absorbed by crops in reactive forms such as nitrate (NO_3_), ammonia (NH_3_), and nitrogen oxides (NOx). These reactive forms can be sustainably produced by soil microbes, but with chemical fertilizers, excessive amounts remain in the soil, flow to the groundwater, and even contribute to the production of greenhouse gases such as nitrous oxide (N_2_O) (Choudhury and Kennedy, 2005; Giles, 2005).

### The Advantage of Biofertilizers in Agriculture

Biofertilizers have been recommended as an alternative to chemical fertilizers in order to avoid the problems caused by chemical fertilizers in agriculture (Mahanty et al., 2017). Biofertilizers are preparations containing living or dormant cells, which have the advantage of growth-promoting functions in crops. This is performed through the production of phytohormones and/or useful substances/biochemicals, thereby enabling the development of ecofriendly and sustainable agriculture (Kumar, 2018). In general, biofertilizers play a significant role in the decomposition of organic matter, which aids mineralization within the soil, consequently increasing the availability of nutrients for plants and improving crop yield (Rodrìguez and Fraga, 1999).

Moreover, the application of biofertilizers can increase the quantity and biodiversity of useful bacteria, such as plant growth-promoting rhizobacteria (PGPR) belonging to *Azotobacter*, *Bacillus*, *Burkholderia*, *Pantoea*, *Pseudomonas*, *Serratia*, and *Streptomyces* (Verma et al., 2019; Gou et al., 2020). Currently, it is believed that the co-evolution of plant–microbe interactions has allowed some of the bacteria to be facultative intracellular endophytes (Bulgarelli et al., 2013), among which are PGPR that have beneficial effects on plants through direct or indirect pathways. For example, some PGPR strains affect plant growth by synthesizing phytohormones, metabolizing them, and/or acting on hormone biosynthesis in plants, while others produce substances that work against soil-borne pathogens (Beneduzi et al., 2012). In the last few decades, as the interest of consumers toward safe agricultural products grew, it became important to exploit beneficial microbes as biofertilizers for use in food safety practices and sustainable crop production (Shi et al., 2011).

### The Role of Microorganisms in Plant Growth

Different microbial species coexist in the soil and have a variety of beneficial effects on plant growth promotion and biological control. These microbes usually improve soil fertility by providing nutrients, such as carbon, nitrogen, phosphorus, potassium, trace elements, vitamins, and amino acids, and making them accessible for plants. In doing so, they promote growth by mediating various activities, such as nitrogen fixation and phosphate and potassium solubilization (Güneş et al., 2014; Bagyalakshmi et al., 2017). These microbes are able to release plant growth-regulating substances, such as phytohormones, and also have a suppressive effect on plant diseases by producing/secreting antibiotics and/or secondary metabolites that work against pathogens (Yoshihisa et al., 1989; Sessitsch et al., 2004; Jung et al., 2006). These microorganisms are known as plant growth-promoting bacteria (PGPB), which generally belong to *Pseudomonas*, *Azospirillum*, *Rhizobium*, and *Bacillus* (Walsh et al., 2001; Esitken et al., 2010; Ahemad and Kibret, 2014).

### Characteristics of Microalgae

In addition to PGPB, some microalgae species have also been used to promote plant growth, yield, and fruit quality (Guo et al., 2020). Microalgae are typically microscopic algae, which range in sizes between micrometers and tens of micrometers, depending on the species. It has been estimated that 200,000–800,000 species exist (Ebenezer et al., 2011), but only around 40,000–50,000 species have been described thus far (Suganya et al., 2016). They are photosynthetic eukaryotes, which do not have roots, stems, or leaves, unlike higher plants, and range from unicellular to multicellular species (Singh and Saxena, 2015). Many species of microalgae have been utilized for the removal of contaminants from wastewater or sewage, as well as the conversion of said sewage into an effluent that can be reused for various purposes. This is because of their ability to absorb and metabolize nutrients and heavy metals in the water such as cadmium, lead, zinc, and copper (Rajamani et al., 2007). Under certain growth conditions, microalgae produce and accumulate large amounts of lipids, proteins, and carbohydrates in the cell. The lipid content of microalgae is higher than that of other biofuels, usually between 20% and 50% of the cell’s dry weight but can reach up to 70% (Duan et al., 2012; Sun et al., 2018). Because of this, they have also been regarded as a suitable candidate for third-generation biofuel feedstock. A variety of studies have been conducted to maximize the potential of microalgae as biofuels, including the applications of genetically modified microalgae; high-density mass culturing; and efficient processes for cultivation, harvest, and extraction (Ghasemi et al., 2012).

In recent years, an increasing amount of research has been conducted to study the effects of microalgal–bacterial co-culture/combination systems for wastewater treatment and biomass production (Ramanan et al., 2016; Qi et al., 2018). However, investigations into microalgal–bacterial co-culture/combination systems for crop production remain largely unexplored. Hence, the current review describes the effects of microalgae and bacteria as biofertilizer agents in vegetable cultivation. Furthermore, it aims to propose the potential of the microalgae–PGPB co-culture/combination system to improve the production and the quality of vegetables.

## Evaluation of Microalgae as Biofertilizers for Vegetable Production

### Effect of Microalgae as Biofertilizers on Crop Cultivation

Many studies have indicated that microalgae are increasingly being employed, not only in bioremediation and biofuel production but also in agriculture. This is because a wide range of bioactive compounds, including plant growth-promoting substances (such as phytohormones), amino acids, carotenoids, and phycobilins, can be produced from microalgae. These compounds contribute to high productivity in agricultural crops by promoting plant growth and conferring resistance against pathogens with minimal environmental costs (Stirk et al., 2013b; Michalak and Chojnacka, 2014).

Microalgal extracts contain phytohormones such as auxin, cytokinin, abscisic acid, ethylene, and gibberellin, which play key roles in the regulation of growth and development. Accordingly, microalgal extracts can be used as renewable sources of plant biostimulation (Stirk et al., 2013a; Romanenko et al., 2015). Auxin is an essential regulator of various plant developmental processes, such as cell division and elongation. Indole-3-acetic acid (IAA) and indole-3-butanoic acid (IBA), the two dominant types of auxins in microalgae, can both stimulate and inhibit the growth and metabolism of higher plants (Hashtroudi et al., 2013). Cytokinins are involved in many physiological processes in plants, including root and shoot development, leaf senescence, nutrient mobilization, and seed germination (Ha et al., 2012), while gibberellins are able to promote cell division, trigger the accumulation of pigments and proteins, and stimulate cell elongation and expansion (Sponsel and Hedden, 2010). Ethylene is a gaseous plant hormone that renders tolerance to abiotic stresses such as drought, low temperatures, and high salinity, as well as biotic stresses such as the penetration of pathogens (Pierik et al., 2006). Thus, phytohormones not only affect plant growth and development but also activate plant defense systems against plant pathogens via interacting/cross-talking networks among them (Checker et al., 2018).

### Various Microalgae Species Used for Important Vegetable Cultivation

Based on the increasing number of studies demonstrating that microalgae have the ability to promote plant growth and defend against plant pathogens, several microalgae species are being used in the cultivation of important vegetables (Table 1). In general, *Chlorella vulgaris*, *Chlorella fusca*, and *Spirulina platensis* have been used for tomato, cucumber, onion, lettuce, and pepper cultivation with a view to promote their production with marketable quality (Kim et al., 2018; Bumandalai, 2019; Rachidi et al., 2020).

**TABLE 1:** Typical cases of microalgal effects on vegetable production.

Vegetable	Microalgae species	Application	References
Tomato	*Nannochloropsis oculata*	Increased contents of sugar and carotenoid in fruits	Coppens et al. (2016)
	*Chlorella vulgaris*	Improved growth of shoot and root	Bumandalai (2019)
	*Arthrospira platensis*, *Dunaliella salina*, and *Porphyridium* sp.	Improved activities of nitrate reductase (NR) and NAD-glutamate dehydrogenase (NAD-GDH) related to nitrogen assimilation and amino acid synthesis in leaves	Rachidi et al. (2020)
	*Chlorella vulgaris* and *Chlorella sorokiniana*	Increased activities of β-1,3-glucanase and phenylalanine ammonia lyase (PAL) linked to defense mechanisms in leaves	Farid et al. (2019)
	*Acutodesmus dimorphus*	Increased number of branches and flowers in plants	Garcia-Gonzalez and Sommerfeld (2016)
Onion	*Spirulina platensis* + cow dung	Improved growth, yield, and content of pigments in leaves and elevated levels of biochemicals and minerals	Dineshkumar et al. (2020)
	*Scenedesmus subspicatus* + humic acid	Promoted root growth at the early developmental stages and increased contents of sugars and proteins in bulbs	Gemin et al. (2019)
Cucumber	*Chlorella vulgaris*	Promoted root growth	Bumandalai (2019)
	*Anabaena vaginicola* and *Nostoc calcicola*	Improved rooting abilities likely affected by indole-3-butyric acid (IBA) and indole-3-acetic acid (IAA)	Shariatmadari et al. (2013)
Eggplant	*Spirulina platensis*	Increased fruit production without significant alterations in the levels of N, P, K, and Na in the leaves, when treated with low concentrations	Dias et al. (2016)
Pepper	*Dunaliella salina* and *Phaeodactylum tricornutum*	Improved salt tolerance during germination by reducing superoxide radicals and lipid peroxidation	Guzmán-Murillo et al. (2013)
Lettuce	*Chlorella vulgaris*	Reduced mineral fertilizer consumption up to 60% by adding living *Chlorella vulgaris* in the nutrient solution	Ergun et al. (2020)
	*Scenedesmus quadricauda*	Increased plant growth and protein content in leaves by activating key enzymes related to N, C, and secondary metabolisms (i.e., phenylalanine ammonia lyase; PAL)	Puglisi et al. (2020)

#### Tomato (*Solanum lycopersicum* L.)

**FIGURE 1:**
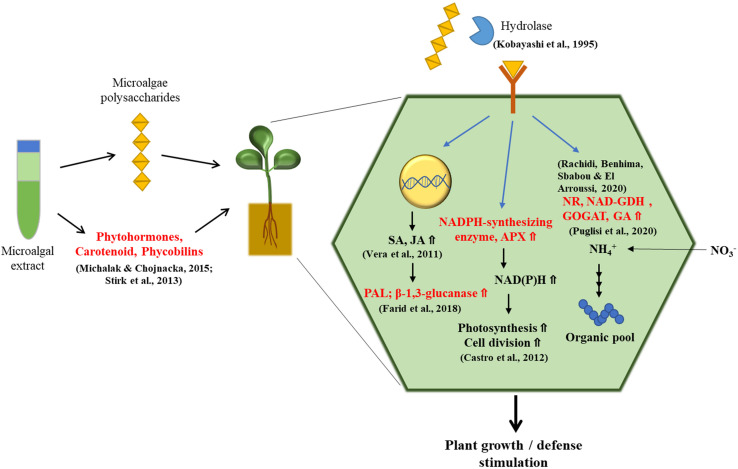
The treatment of microalgae extracts stimulates plant growth and defense system. Microalgal extracts contain many plant growth-promoting compounds such as polysaccharides, phytohormones, carotenoids, and phycobilins, which have the ability to stimulate the plant growth and defense system. Large polysaccharides from microalgal extracts are broken down into smaller fragments of oligosaccharides by hydrolytic enzymes. Oligosaccharides are perceived by plant’s membrane receptor and have a significant stimulatory effect on the plant growth by regulating activities of enzymes such as nitrate reductase (NR) and NAD-glutamate dehydrogenase (NAD-GDH) related with nitrate assimilation. Moreover, polysaccharides can increase activity of NADPH-synthesizing enzymes, ascorbate peroxidase (APX), and the amount of ascorbate (AsA), which are associated with photosynthesis, fundamental cellular metabolism, and cell cycle. Defense pathways are also stimulated by polysaccharides in plant cells: polysaccharide treatment upregulates the expression of genes involved in salicylic acid (SA) and jasmonic acid (JA) signaling pathways, resulting in increased activities of β-1,3-glucanase and phenylalanine ammonia lyase (PAL) linked to plant defense system.

Tomato is the most popular home garden vegetable. It is a rich source of vitamins, minerals, and flavonoids such as quercetin (Nicola et al., 2009). Strategies for improving the productivity and nutritional quality of tomatoes are of great interest to producers (Dorais et al., 2008). In tomato fruits, treatment with *Nannochloropsis oculata* has been found to induce 33% and 36% higher levels of sugar and carotenoid content, respectively, compared to those treated with inorganic fertilizer under greenhouse conditions (Coppens et al., 2016). It was also found that among young tomato plants grown in phytotrons, the number of nodes, dry weight, and length of shoots had significantly increased from treatment with polysaccharide extracts from *Arthrospira platensis*, *Dunaliella salina*, and *Porphyridium* sp. when compared to the untreated control. Tomato plants treated with polysaccharide extracts also showed an increase in the activities of nitrate reductase (NR) and NAD-glutamate dehydrogenase (NAD-GDH), key enzymes for nitrogen assimilation and amino acid synthesis, as well as phenylalanine ammonia lyase (PAL) and β-1,3-glucanase, which activate plant defenses against pathogens (Kobayashi et al., 1995; Vera et al., 2011; Chen et al., 2017; Farid et al., 2019; Rachidi et al., 2020). Microalgal polysaccharides can elevate the activity of NADPH-synthesizing enzymes, shifting conditions to be more conducive to reduction in the intracellular redox state, which may favor photosynthesis and cell division. In addition, the levels of ascorbate (AsA) content and ascorbate peroxidase (APX) activity, which play central roles in photosynthesis and abiotic stress tolerance, have been shown to increase in polysaccharide-treated plants (Castro et al., 2012; Smirnoff, 2018; Chanda et al., 2019; Figure 1). Moreover, Garcia-Gonzalez and Sommerfeld (2016) assessed the properties of the microalgae *Acutodesmus dimorphus* as a biofertilizer and/or biostimulant. Under greenhouse conditions, foliar application of the algal extract at a concentration of 3.75 g ml^–1^ on tomato plants caused an increase in the number of branches and flowers per plant (Garcia-Gonzalez and Sommerfeld, 2016).

#### Onion (*Allium cepa* L.)

Onion is one of the most economically important vegetable crops consumed primarily because of its ability to enhance the flavor of other foods (Kandoliya et al., 2015). Field experiments were performed at the experimental farm of Annamalai University, India, in 2016–2017 to determine the efficacy of microalgae, such as *C. vulgaris* and *S. platensis*, as biofertilizers on onion plants. Treatment comprising of cow dung with *S. platensis* on onion plants resulted in higher amounts of micro- and macronutrients available in the soil, including nitrogen, phosphate, potassium, zinc, and manganese. There was also an increase in the levels of biochemicals in the onion, such as total soluble sugars, total phenols, and free amino acids, along with improved growth parameters, when compared to the untreated control. In addition to this, higher amounts of minerals were observed in onion plants treated with “cow dung + *S. platensis*” and “cow dung + *C. vulgaris*” (Dineshkumar et al., 2020). Furthermore, compared to the untreated control, treatment with a mixture of *Scenedesmus subspicatus* and humic acid synergistically increased the onion root length by 39% and the concentration of soluble proteins by 37% (Gemin et al., 2019).

#### Cucumber (*Cucumis sativus* L.)

Cucumber is also an important vegetable crop (Huang et al., 2009) that is cultivated in many countries within both temperate and tropical zones (Tatlioglu, 1993). When vegetable crops such as cucumber, tomato, and squash were treated with *Anabaena vaginicola* and *Nostoc calcicola*, they showed increased growth factors when compared to the untreated control. These growth factors included root length, fresh and dry weight of roots, and plant height. Auxins such as IBA, which are involved in root development in plants, were also shown to be available with this treatment in the range of 1.275–2.958 μg g^–1^ dry weight with a trace amount of IAA in microalgal cells (Shariatmadari et al., 2013).

#### Eggplant (*Solanum melongena* L.)

Eggplant is ranked among the top 10 vegetables in terms of oxygen radical absorbance capacity due to its high content of total phenolics (Cao et al., 1996). Dias et al. (2016) conducted field and laboratory experiments to evaluate the growth, yield, and postharvest quality of eggplants with different concentrations of *S. platensis* solutions for foliar application at the Centro de Ciências e Tecnologia de Alimentos (CCTA), Brazil. This experiment was performed between October 2014 and January 2015. The results revealed that the number of fruits significantly increased in plants treated with low concentrations of this treatment (10, 15, 25, and 35 g l^–1^). This is likely due to the greater abundance of polypeptides, amino acids, and hormones in the microalgal species acting as plant growth promoters when compared to the levels in the untreated control (Dias et al., 2016).

#### Pepper (*Capsicum annuum* L.)

Pepper is a popular commercial vegetable and spice crop that is valued for its fruit color, flavor, pungency, and nutrient content (Kumar et al., 2006). Treatment with extracts of *D. salina* increased the germination rate by 69% and the root length of bell peppers by 24% in 25 mM NaCl. Meanwhile, *Phaeodactylum tricornutum* treatment was found to reduce the production of superoxide radicals and lipid peroxidation triggered by salt stress, when compared to the untreated control (Guzmán-Murillo et al., 2013).

#### Lettuce (*Lactuca sativa* L.)

Treatment of lettuce seedlings with *Scenedesmus quadricauda* extract promoted plant growth and induced the accumulation of chlorophyll, carotenoids, and total protein content in the plant cell. In addition, the leaf dry weights were positively affected by the treatment with *S. quadricauda* extract, reaching an increase of approximately 26% when compared to the untreated control. From a metabolic point of view, the treated leaves revealed increased enzyme activity levels of glutamate synthase (GOGAT), glutamine synthase (GS), citrate synthase (CS), malate dehydrogenase (MDH), and PAL, which are key enzymes associated with nitrogen (Gupta et al., 2012), carbon (Schiavon et al., 2008), and phenylpropanoid metabolism (Hyun et al., 2011). This suggested that the positive effect on the growth of lettuce most likely occurs through the stimulation of the metabolic pathways of carbon, nitrogen, and phenylpropanoid (Puglisi et al., 2020).

## Evaluation of Bacteria as Biofertilizers for Vegetable Production

### The Effect of Bacteria as Biofertilizers on Crop Cultivation

Bacteria are a major class of microorganisms that function as decomposers and recyclers in the soil. In doing so, these microbes contribute to the processes of nutrient cycling, energy flow, and bioconversion in the ecosystem. Most agricultural production systems are dependent on soil bacterial biomass pools, which facilitate quick responses to diverse environmental changes (Pankhurst et al., 1996). Microbial inoculums called effective microorganisms (EM), containing mixed cultures of beneficial and naturally occurring microorganisms, can increase the microbial diversity of the soil ecosystem. They consist mainly of lactic acid bacteria, photosynthetic bacteria, yeast, *Actinomyces*, and fermenting fungi (Balogun et al., 2016). Among these effective microorganisms, PGPB form specific symbiotic relationships with plants and directly promote plant growth by facilitating resource acquisition and/or modulating plant hormone levels (Glick, 1995). The application of PGPB to vegetable cultivations can prevent the excessive use of chemical fertilizers by up to 30%, thereby reducing production costs and pollution (Geries and Elsadany, 2021). PGPB treatments also have the ability to improve host plant defenses against soil-borne pathogens by producing antibiotics such as 2,4-diacetylphloroglucinol (2,4-DAPG), pyoluteorin (PLT), pyrrolnitrin, and phenazine-1-carboxylate (Bangera and Thomashow, 1999; Duffy and Défago, 1999).

### Various Bacterial Species Used for Vegetable Cultivation

It has been adequately demonstrated that many species of bacteria are able to promote the growth and development of vegetables and control pathogens through various mechanisms, one of which include the production/release of inhibitory substances, allowing target crops to be disease resistant (Table 2). Representative commercially available bacterial strains are of the genus *Bacillus* spp.; these include *B. amyloliquefaciens*, *B. subtilis*, *B. cereus*, *B. licheniformis*, and *B. pumilus*, which produce various compounds for the biocontrol of plant pathogens and the growth promotion of vegetables such as tomato, cucumber, onion, lettuce, and pepper (Gutiérrez-Mañero et al., 2001; Compant et al., 2005; Cawoy et al., 2011; Yuan et al., 2012; Nie et al., 2017). Silo-Suh et al. (1994) reported that *B. cereus* UW85 suppresses the damping-off disease caused by *Phytophthora medicaginis* in alfalfa through the production of two fungistatic antibiotics, zwittermicin A and kanosamine (Silo-Suh et al., 1994).

**TABLE 2:** Typical cases of bacterial effects on vegetable production.

Vegetable	Bacteria species	Application	References
Tomato	*Serratia liquefaciens* and *Pseudomonas putida*	Induced systemic resistance against the fungal leaf pathogen *Alternaria alternata* in tomato by producing *N*-*acyl*-L-homoserine (AHL) lactone	Schuhegger et al. (2006)
	*Pantoea agglomerans* and *Burkholderia anthina*	Increased plant height, root length, shoot and root dry weight, phosphorous uptake level, and the available phosphorus content of soil	Walpola and Yoon (2013)
	*Bacillus amyloliquefaciens*	Suppressed bacterial wilt disease by reducing the population of *Ralstonia solanacearum*	Huang et al. (2014)
	*Bacillus circulans*	Stimulated seedling growth by increasing nutrient uptake parameters	Mehta et al. (2015)
Onion	*Azotobacter chroococcum*, *Bacillus subtilis*, and *Pseudomonas fluorescens*	Produced indole-3-acetic acid (IAA) and siderophores and improved growth and yield with higher solubilization of tricalcium phosphate (TCP)	Čolo et al. (2014)
	*Bacillus subtilis*	Inhibited the growth of *Setophoma terrestris*, a causal agent of pink root disease	Albarracín Orio et al. (2016)
Cucumber	*Pseudomonas corrugate* and *Pseudomonas aureofaciens*	Inhibited root and crown rot caused by *Pythium aphanidermatum* by stimulating the activities of defense enzymes in the root tissue	Chen et al. (2000)
	*Bacillus subtilis*	Improved growth and yield by reducing losses caused by *Pythium* root rot	Utkhede and Koch (1999)
Lettuce	*Bacillus amyloliquefaciens*	Alleviated the disease severity of bottom rot caused by *Rhizoctonia solani*.	Chowdhury et al. (2013)
Pepper	*Bacillus licheniformis* and *Bacillus subtilis*	Produced auxins, antifungal β-glucanases, and siderophores; stimulated seed germination; and promoted the growth of vegetative organs such as root, stem, and leaf	Lim and Kim (2009)

In addition, *Serratia liquefaciens* and *Pseudomonas putida* are known to generate *N-acyl*-L-homoserine lactone (AHL) signaling molecules, which enhance the systemic resistance of tomato plants against the leaf fungal pathogen, *Alternaria alternata* (Schuhegger et al., 2006). Recently, it was shown that two phosphate-solubilizing bacteria (PSB), *Pantoea agglomerans* and *Burkholderia anthina*, contributed to improved growth traits of tomato plants with a higher level of phosphorous content in the soil, when compared to the untreated control, under greenhouse conditions (Walpola and Yoon, 2013). The ability of *Azotobacter chroococcum* and *Pseudomonas fluorescens* to improve vegetative growth and yield in onion production through the production of IAA, siderophores, and the solubilization of tricalcium phosphate (TCP) has also been demonstrated (Tarakhovskaya et al., 2007; Čolo et al., 2014).

## Relationship Between Microalgae and Bacteria

### Microalgae–Bacteria Interactions

In natural environments, microalgae and bacteria coexist and interact with each other. As a result, they demonstrate both beneficial (Unnithan et al., 2014) and harmful/toxic relationships (Doucette, 1995). The relationship between microalgae and bacteria is greatly dependent on the species and the environmental conditions (Doucette, 1995; Croft et al., 2005; Mujtaba and Lee, 2016). In actuality, both microalgae and bacteria can produce growth factors, and/or exotoxins, that promote and/or inhibit growth and development. In the beneficial relationship, microalgae enhance bacterial growth by providing photosynthetic oxygen and dissolved organic matter such as organic carbon, calcium carbonate, and 2,3-dihydroxypropane-1-sulfonate (DHPS) (Wolfaardt et al., 1994; Borde et al., 2003; Cooper and Smith, 2015). Generally speaking, the photosynthetic oxygen produced by microalgae or cyanobacteria (blue-green algae) is used as an electron acceptor in the bacterial degradation of organic matter. In turn, bacteria support photoautotrophic growth of their partners by providing carbon dioxide and other stimulatory means (Subashchandrabose et al., 2011). In a similar way, bacteria are also able to offer a selective advantage to microalgae for enhanced growth by providing micronutrients such as B-vitamins. These vitamins act as co-factors that are required for enzyme activity in the central cellular metabolism (Croft et al., 2005). Moreover, microalgae can acquire nutrients such as inorganic carbon, nitrogen, phosphorus, and sulfate generated from organic matter, through the activities of extracellular bacterial enzymes. However, in harmful/toxic relationships, microalgae can inhibit bacterial activity by releasing antibacterial metabolites and increasing the pH, the dissolved oxygen concentration, and the temperature of the culture medium (Naviner et al., 1999; Schumacher et al., 2003; Ribalet et al., 2008).

**FIGURE 2:**
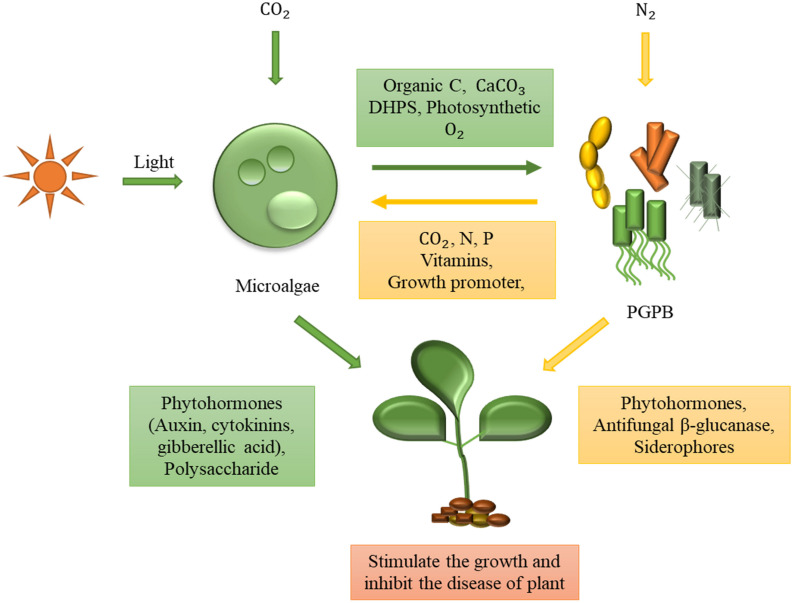
Symbiotic interactions between microalgae and plant growth-promoting bacteria (PGPB) for sustainable cultivation of plants. Microalgae and PGPB in the symbiotic relationship cooperate with each other by efficient exchange of nutrients. Microalgae supply photosynthetic oxygen, organic carbon, calcium carbonate, and 2 3-dihydroxypropane-1-sulfonate (DHPS) to bacteria in exchange for micronutrients (i.e., vitamins) and macronutrients (i.e., nitrogen and phosphorus). Both microalgae and bacteria can enhance plant growth by producing phytohormones and other growth stimulants. Moreover, they are also able to inhibit plant diseases by using their distinct disease-suppressive mechanisms.

Both microalgae and PGPB have the ability to promote plant growth by producing polysaccharides and phytohormones, such as auxin and cytokinin. Furthermore, they can prevent plant diseases by stimulating defense systems and secreting antifungal enzymes and antibiotics (Figure 2; Najdenski et al., 2013; Stirk et al., 2013b; Walpola and Yoon, 2013; Michalak and Chojnacka, 2014; Cordero et al., 2016).

### Reciprocal Influence During the Co-culture/Combination of Microalgae and Bacteria

As microalgae have been widely used in various industries, extensive studies have shown that when microalgae and bacteria are co-cultured, there is an increase in microalgal productivity in the production of useful substances such as total lipids, carbohydrates, and chlorophylls (Table 3).

**TABLE 3:** Examples of microalgae–bacteria interactions in co-culture/combination.

Microalgae	Bacteria	Comments	References
*Chlorella sorokiniana*	*Azospirillum brasilense* and *Bacilus pumilus*	Two plant growth-promoting bacteria (PGPB) remotely enhanced the growth of the microalgae with elevated amounts of total lipids, carbohydrates, and chlorophyll a in the microalgal cells	Amavizca et al. (2017)
	Bacterial strain CSSB-3 (98.6% identical to the 16S rDNA gene sequence of *Microbacterium trichotecenolyticum*)	Promoted the growth of *C. sorokiniana* in the mixed culture with CSSB-3 under a photoautotrophic condition	Watanabe et al. (2005)
	*Azospirillum brasilense*	*Azospirillum brasilense* increased *C. sorokiniana* growth through a variety of mechanisms, including the production of IAA	Peng et al. (2020)
*Chlorella vulgaris*	*Azospirillum brasilense*	Increased pigment and lipid contents, lipid variety, and cell and population size of the microalgae *Chlorella* spp.	de-Bashan et al. (2002)
*Amphidinium operculatum*	*Halomonas* sp.	*Amphidinium* acquires vitamin B_12_ through a direct interaction with *Halomonas* sp.	Croft et al. (2005)
*Tetraselmis chuii*	*Muricauda* sp.	*Muricauda* significantly promoted the growth of *T. chuii* and *C. fusiformis*, but drastically inhibited the growth of *N. gaditana*.	Han et al. (2016)
*Cylindrotheca fusiformis*			
*Nannochloropsis gaditana*			

Amavizca et al. (2017) demonstrated that two PGPB strains, *Azospirillum brasilense* Cd and *B. pumilus* ES4, have similar effects on the growth of the green microalga *Chlorella sorokiniana* UTEX 2714, without any form of physical contact between them. The two PGPB remotely enhanced the growth rate of microalgae up to six-fold and induced an increase in the total amounts of lipids, carbohydrates, and chlorophyll *a* in the microalgal cells. These beneficial effects have been ascribed to the volatile compounds produced by the bacteria, which include CO_2_ (Amavizca et al., 2017). In addition, plant growth-promoting bacteria, such as *A. brasilense*, have the potential to significantly increase *C. sorokiniana* growth rates through a variety of mechanisms, including the production of IAA (Peng et al., 2020). Croft et al. (2005) reported that bacterial strains of *Halomonas* sp. have a growth-enhancing effect on the microalga *Amphidinium operculatum* through the provision of vitamin B_12_. In addition to this, the presence of algal extracts also leads to the promotion of bacterial growth with upregulated vitamin B_12_ biosynthesis, displaying positive reciprocity (Croft et al., 2005). Sometimes, a single bacterial strain can induce different effects on the growth and proliferation of distinct microalgae. For example, *Muricauda* sp. was found to promote the growth of *Tetraselmis chuii* and *Cylindrotheca fusiformis*, but drastically inhibited the growth of *Nannochloropsis gaditana* (Han et al., 2016).

## Co-Culturing/Combination of Microalgae–Bacteria

### Microalgae–Bacteria Co-culture for Wastewater Treatment and Biomass Production

The co-culture system of bacteria and microalgae has been mainly used for wastewater treatment and biomass production. Mujtaba et al. (2017) investigated the efficiency of nutrient removal (i.e., ammonium, phosphate, etc.) and the reduction of chemical oxygen demand (COD) from wastewater, using the symbiotic co-culture of *P. putida* and immobilized *C. vulgaris*. In general, symbiotic co-culture systems facilitate the simultaneous removal of a greater variety of nutrients from wastewater compared to monocultures (Mujtaba et al., 2017). Ogbonna et al. (2000) found that it is impossible to remove all nutrients such as acetate, propionate, ammonia, nitrate, and phosphorus from wastewater at once using monocultures of *Rhodobacter sphaeroides*, *C. sorokiniana*, and *S. platensis*, whereas these nutrients can all be removed simultaneously by using the co-culture system (Ogbonna et al., 2000). Moreover, the symbiotic co-culture of *A. brasilense* and *Scenedesmus* sp. has been successfully applied to biofuel production with higher biomass volume. This indicates that the symbiotic co-culture has the potential to increase microalgal colony size, and the fatty acid content inside biofuels, in nitrogen-deficient media (Contreras et al., 2019). The combination of cyanobacteria/microalgae and bacteria can more efficiently detoxify organic and inorganic pollutants and remove nutrients from wastewater compared to using either of them alone.

Soils and aquatic systems contaminated by heavy metals have also become a serious issue in crop production because of the risks associated with food contamination. Microalgae have the ability to detoxify and volatilize heavy metals via microalgal metabolism and high levels of metal binding (Yu et al., 1999). Microalgae form metal-binding peptides (organometallic complexes) such as class III metallothionein (MtIII) (Perales-Vela et al., 2006), which facilitate appropriate control of the cytoplasmic concentration of heavy metals, thereby preventing or neutralizing the potential toxicity caused by heavy metals (Kaplan, 2013; Priya et al., 2014). The application of microalgae, which can bioabsorb and biotransform arsenate (As) in rice fields, reduces the availability of As to plants, thereby rendering the food grains safe for human consumption (Debnath and Bhadury, 2016). In the case of the algal–bacterial synergistic interactions, a higher removal efficiency of heavy metals was achieved with the addition of bacterial inoculum, which enhanced algal growth with additional CO_2_ and organic compounds provided by the bacteria (Unnithan et al., 2014; Mubashar et al., 2020). Furthermore, algae can also be recycled as biofertilizing agents (Guo et al., 2020).

### Potential of Microalgae–Bacteria Co-cultures/Combination for Vegetable Cultivation

There are two distinct application methods for the simultaneous use of microalgae and bacteria, one is co-culturing the microbes from the beginning and the other is preparing a mixture containing microalgae/microalgal extract and bacteria gained from each pure culture (combination).

The combined application of specific bacteria, which are growth promoters in plants and/or biocontrol agents against plant pathogens, can lead to the synergy necessary for ideal vegetable cultivation. When *Pantoea ananatis* and *P. fluorescens* (CPP-2) were co-cultured, IAA production and phosphate solubilization were higher than those from either strain alone. Additionally, the co-culture of CPP-2 showed promotion effects on root and shoot elongation of pea (*Pisum sativum*) plants when compared to the culture of an individual strain (Anwar et al., 2019). Moreover, the combination of *P. putida* WCS358 and RE8 was shown to enhance the suppression of *Fusarium* wilt in radish by approximately 50%, when compared to the untreated control, while that of the single-strain treatments was reduced by 30%. Even when one strain failed to suppress disease with a single application, the combination treatment still exhibited a suppressive effect against the disease. This implies that the reinforced resistance, brought on the combination application, is likely due to the additive/combinational effect of different disease-suppressive mechanisms (Boer et al., 2003).

Even though the application of microorganisms, via co-culture/combination treatment, is more effective for vegetable production (Minuto et al., 1993; Raupach and Kloepper, 1998), the use of a single microbial species for vegetable cultivation is commonly observed in practical situations. Although the activity of one microbial species is relatively narrow in scope from a practical point of view, it is easier to obtain related patents and/or safety certificates for the use of a single microbial species in agricultural and commercial sectors with obvious effects in a short period of time. In addition to this, the application of a single microbial species makes the processes of cultivation, harvesting, and extraction simpler and easier (Sylvia et al., 2005). However, in the case of co-culture/combination, the ability to suppress plant pathogens and promote plant growth is much more effective than that of the monoculture system because the microbes in the co-culture/combination system promote the growth of plants and/or prevent pathogens by different mechanisms (Spadaro and Gullino, 2005).

Several important practical examples of mixed treatments of cyanobacteria/microalgae–bacteria have been reported for crop cultivation (Table 4).

**TABLE 4:** Examples of co-inoculation of microalgae–bacteria in agriculture.

Crop	Microalgae/cyanobacteria	Bacteria	Application	References
Rice	*Anabaena laxa, Anabaena* sp., and *Anabaena oscillarioides*	*Providencia* sp., *Brevundimonas* sp., and *Ochrobactrum* sp.	Enhanced carbon sequestration and plant growth in treatments involving a combination of bacterial and microalgal strains	Prasanna et al. (2012)
Lettuce	*Chlorella vulgaris*	*Bacillus licheniformis*, *Bacillus megatherium*, *Azotobacter* sp., *Azospirillum* sp., and *Herbaspirillum* sp.	Increased the plant weight and total carotenoid content especially under stress conditions during summer	Kopta et al. (2018)
Common bean	*Anabaena cylindrica*	*Rhizobium tropici*, *Rhizobium freirei*, and *Azospirillum brasilense*	Promoted plant growth parameters and grain production by 84% in plants inoculated with *Rhizobium* + *Azospirillum* + *Anabaena*	Horácio et al. (2020)
Maize	*Anabaena cylindrica*	*Azospirillum brasilense*	Increased yield performance of maize hybrid in Londrina and Faxinal	Gavilanes et al. (2020)
Onion	*Spirulina platensis*	*Pseudomonas stutzeri*	Enhanced plant growth, productivity, and bulb quality and reduced the production cost in treatments involving the combined treatment of *S. platensis* extract and nitrogen-fixing *P. stutzeri*	Geries and Elsadany (2021)

In 2012, the effects of combined treatments of cyanobacterial strains—CR1, CR2, and CR3 (*Anabaena laxa*, *Anabaena* sp., and *Anabaena oscillarioides*)—and bacterial strains—PR3, PR7, and PR10 (*Providencia* sp., *Brevundimonas* sp., and *Ochrobactrum* sp.)—on rice crop yield and C-N sequestration in soil were first reported in a pot experiment (Prasanna et al., 2012).

The synergistic efficacy of the combination of freshwater algae (*C. vulgaris*) and plant growth-promoting bacteria (*B. licheniformis*, *Bacillus megatherium*, *Azotobacter* sp., *Azospirillum* sp., and *Herbaspirillum* sp.) on yields and nutritional values of leaf and romaine lettuces has also been observed. The combined application of microalgal–bacterial preparation led to a significant increase of the romaine and leaf lettuce weight by 12.9% and 22.7%, respectively. Of note, total carotenoids in amounts 26.7% higher than in the controls were detected in the treated romaine lettuce under stress conditions during summer (Kopta et al., 2018).

Recently, Horácio et al. (2020) evaluated the effects of co-inoculation with a diazotrophic cyanobacterium (*Anabaena cylindrica*, Ana), *Rhizobium* (*R. tropici* + *R. freirei*, Riz), and *A. brasilense* (Azo) on the development of the common bean under greenhouse conditions. Grain production in the plants co-inoculated with Ana + Riz + Azo and fertilized with N (100 kg N ha^–1^) was 84.4% and 86.3% higher than that of untreated controls, respectively. This indicates that N fertilization can be replaced by co-inoculation with selected cyanobacterial–bacterial strains (Horácio et al., 2020). In addition, Gavilanes et al. (2020) conducted two field experiments to assess the efficacy of co-inoculation of *A. cylindrica* with *A. brasilense* on the yield performance of four maize cultivars in two locations (Londrina and Faxinal in Paraná, Brazil). They found that the co-inoculation of *A. cylindrica* and *A. brasilense* resulted in a yield that increased by 9% (967 kg ha^–1^) in Londrina and 23% (1,744 kg ha^–1^) in Faxinal, compared to the uninoculated control (Gavilanes et al., 2020).

It was also found that the growth and productivity of onion plants with the combination treatment were promoted when compared to those with either single-agent treatment. This supports the result of biochemical analyses that state that extracts of both *S. platensis* and *Pseudomonas stutzeri* possess bioactive compounds such as HCN, NH_3_, IAA, and amino acids that have stimulatory effects on plant growth and quality (Geries and Elsadany, 2021).

Additionally, after culturing *C. fusca* and *B. amyloliquefaciens* cc178 separately, they were combined in a ratio of 2:1 and used to irrigate tomato roots. It was found that rooting of fine roots was promoted and the plant growth, yield, and soluble liquid sugar content in fruits had significantly increased with the combination treatment when compared to each single treatment (unpublished data). Therefore, the combined application of microalgae and PGPB can exert beneficial effects on the yield and quality of vegetable crops.

## Conclusion and Future Directions

Microalgae and bacteria have received great interest as biofertilizers in ecofriendly vegetable production. Until now, monoculture systems using certain agricultural microorganisms have been highlighted to improve the yield and quality of agricultural products. However, co-culture/combination systems of microorganisms can be more effective in enhancing microbial diversity in the soil, resistance to plant diseases, and productivity of vegetable crops. Therefore, further investigations to uncover the molecular mechanisms underlying the effect of microalgae–bacteria co-culture/combination on vegetable growth, and/or plant disease suppression, will be necessary for the extension of sustainable agriculture.

## Author Contributions

All authors listed have made a substantial, direct and intellectual contribution to the work, and approved the final version of the manuscript for publication.

## Conflict of Interest

The authors declare that the research was conducted in the absence of any commercial or financial relationships that could be construed as a potential conflict of interest.
